# Drug rash induced by levothyroxine and oral desensitization

**DOI:** 10.1186/1939-4551-8-S1-A21

**Published:** 2015-04-08

**Authors:** Sérgio Duarte Dortas, Franklin Moreira De Araujo, Cintia Ribas Souza, Eduardo Micmacher, Soloni Afra Pires Levy, Augusto Tiaqui Abe, Augusto Tiaqui Abe, Alfeu Tavares França

**Affiliations:** 1 Universidade Iguaçu, Brazil; 2 Hospital Universitário Clementino Fraga Filho Hucff-Ufrj, Brazil; 3 Hospital Geral De Nova Iguaçu, Brazil; 4 Hospital São Zacharias, Brazil

## Background

Synthetic Thyroxine (T4) is the treatment of choice for the correction of hypothyroidism.

## Methods

We report a case of drug rash induced by levothyroxine and currently oral desensitization.

A 31 year-old woman had been diagnosed as Hashimoto´s Thyroiditis. She was having levothyroxine for 9 years when she begun to present bilateral eyelids maculopapular rash. Blood exams and physical examination were normal except for the eyelids´ rash.

As she stopped using levothyroxine, the rash has disappeared.

Her endocrinologist prescribed her other brands of levothyroxine, and the patient reported the same reaction.

## Results

Therefore, we performed an oral desensitization with multiple doses of the drug preparation. The procedure was started at a dose of 1,00µg. Every 30 minutes the dose was increased until a total dose of 127,00µg.

After 2 months, she currently tolerates 100,00µg/d.

## Conclusions

It is unusual that biological substances induce allergic reactions when given exogenously.

Once it happens, drug desensitization can be performed at a hospital by experienced professionals with satisfactory response.

## Consent

Written informed consent was obtained from the patient for publication of this abstract and any accompanying images. A copy of the written consent is available for review by the Editor of this journal.

